# Do Glucagon-Like Peptide-1 Receptor Agonists Sabotage Fat Grafts?: A Scoping Review of Glucagon-Like Peptide-1 Receptor Agonist Effects on Adipocyte Biology and Implications for Autologous Fat Transfer

**DOI:** 10.1093/asj/sjag108

**Published:** 2026-06-01

**Authors:** Xavier Chalhoub, Zhi Yang Ng

## Abstract

The rapid adoption of glucagon-like peptide-1 receptor agonists (GLP-1 RAs) for weight management has created an unprecedented overlap with aesthetic surgery. Millions of patients experiencing GLP-1-mediated weight loss now present with facial volume depletion, soft tissue deflation, and contour irregularities; conditions for which autologous fat grafting remains the gold-standard solution. However, the fundamental biology of GLP-1 receptor agonism may be inherently antagonistic to the mechanisms upon which fat graft survival depends. This scoping review, conducted in accordance with the PRISMA extension for Scoping Reviews (PRISMA-ScR), synthesizes current preclinical and clinical evidence on the effects of semaglutide, liraglutide, tirzepatide, and the emerging triple agonist retatrutide on adipocyte metabolism, adipose-derived stem cell (ASC) function, and tissue revascularization, and maps these effects onto established models of fat graft take. We identify multiple potential interference points, including GLP-1-mediated adipocyte browning and thermogenic activation with upregulation of uncoupling protein 1 (UCP1) and mitochondrial uncoupling, enhanced lipolysis through adipose triglyceride lipase and hormone-sensitive lipase (HSL) upregulation, suppression of white adipogenic differentiation in ASCs with preferential commitment toward thermogenic beige lineages, and modulation of inflammatory and angiogenic signaling during the critical revascularization window. The growing off-label use of retatrutide in bodybuilding communities introduces additional concerns through glucagon receptor-mediated lipolysis and thermogenesis. Despite the strong mechanistic rationale, no clinical or preclinical studies have directly examined fat graft outcomes in patients receiving incretin-based therapies. We propose a framework for future investigation and offer preliminary, mechanism-based clinical considerations regarding perioperative GLP-1 RA management in fat transfer patients. These recommendations should be understood as hypothesis-generating rather than evidence-based guidelines.

**Level of Evidence: 1 (Therapeutic)**  
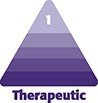

The landscape of aesthetic surgery is being reshaped by the widespread adoption of glucagon-like peptide-1 receptor agonists (GLP-1 RAs). Since the approval of semaglutide (Novo Nordisk, Bagsværd, Denmark) for chronic weight management in 2021, followed by tirzepatide (Eli Lilly and Company, Indianapolis, IN) in 2023, prescriptions for incretin-based weight loss therapies have grown exponentially.^1^ The American Society of Plastic Surgeons identified “GLP-1 makeovers” as a leading aesthetic surgery trend for 2026, reflecting a surge in patients presenting with the sequelae of rapid, pharmacologically mediated weight loss: facial volume depletion (“Ozempic face”), skin laxity, and body contour irregularities.^2,3^

Autologous fat grafting has emerged as a preferred solution for many of these concerns, with approximately 80% of plastic surgeons reporting regular use of the technique.^4^ In facial rejuvenation, fat transfer addresses the hollowed cheeks, deepened nasolabial folds, and periorbital volume loss characteristic of GLP-1-mediated facial deflation.^3^ In body contouring, fat grafting complements skin-tightening procedures to restore natural contours following massive weight loss.^5^ The irony is striking: the very pharmacology that creates the demand for fat grafting may simultaneously undermine its success.

Preclinical and early clinical evidence suggests that GLP-1 RAs exert effects on adipocyte biology that extend beyond appetite suppression. In human subcutaneous adipocyte cultures and biopsy specimens, semaglutide has been shown to upregulate uncoupling protein 1 (UCP1, a mitochondrial protein that converts stored energy to heat) and mitochondrial biogenesis, inducing a “browning” program that shifts white adipocytes toward a thermogenic, energy-dissipating phenotype.^6,7^ In vitro, liraglutide (Novo Nordisk) treated human adipocytes develop smaller lipid droplets and higher expression of lipolytic enzymes including adipose triglyceride lipase (ATGL, the enzyme initiating fat breakdown) and hormone-sensitive lipase (HSL, which continues the process of triglyceride hydrolysis).^8^ Meanwhile, retatrutide (LY3437943; Eli Lilly and Company), a triple GLP-1/GIP/glucagon receptor agonist, achieved 28.7% mean body weight reduction in phase 3 trials and is already circulating in bodybuilding underground markets despite lacking regulatory approval.^9,10^

Fat graft survival depends on a delicate interplay between adipocyte viability, ASC function, and recipient-site revascularization.^4,11^ Each of these mechanisms is potentially compromised by incretin-based therapies. Yet, to date, no clinical or preclinical study has directly examined fat graft outcomes in patients receiving GLP-1 RAs; we found no reports comparing fat graft retention in patients taking these drugs vs controls. This scoping review, following PRISMA-ScR guidelines, aims to bridge this critical knowledge gap by synthesizing the available evidence on GLP-1 RA effects on adipose tissue biology, mapping these effects onto established models of fat graft survival, and proposing a framework for future investigation. A glossary of key biochemical and cellular terms is provided in Supplemental Table 1, available online at https://doi.org/10.1093/asj/sjag108, to assist readers less familiar with molecular biology terminology.

## METHODS

### Search Strategy

This scoping review was conducted in accordance with the Preferred Reporting Items for Systematic Reviews and Meta-Analyses extension for Scoping Reviews (PRISMA-ScR) guidelines. The literature search encompassed studies published from database inception through January to February 2026. The search was designed and executed by X.C. Search results were independently screened by X.C. and Z.Y.N., with disagreements regarding study eligibility resolved by consensus discussion between the two reviewers. A systematic literature search was performed across PubMed/MEDLINE, Embase, and Google Scholar. Three independent search strategies were employed: Search 1 (GLP-1 RA adipose biology): (“GLP-1” OR “semaglutide” OR “liraglutide” OR “tirzepatide” OR “retatrutide” OR “incretin”) AND (“adipose” OR “adipocyte” OR “browning” OR “UCP1” OR “lipolysis” OR “adipose-derived stem cell” OR “ASC”), yielding 847 results; Search 2 (fat graft biology): (“fat graft” OR “fat transfer” OR “lipotransfer” OR “lipofilling”) AND (“survival” OR “retention” OR “angiogenesis” OR “stem cell”), yielding 1243 results; and Search 3 (direct intersection): (“GLP-1” OR “semaglutide” OR “tirzepatide” OR “retatrutide”) AND (“fat graft” OR “fat transfer” OR “lipotransfer”), yielding 0 results. An additional gray literature search was performed in Google Scholar, ClinicalTrials.gov, and preprint servers (medRxiv, bioRxiv) using equivalent terms. Reference lists of all included articles were hand-searched for additional relevant studies. The search flow is presented in Figure 1.

**Figure 1.**
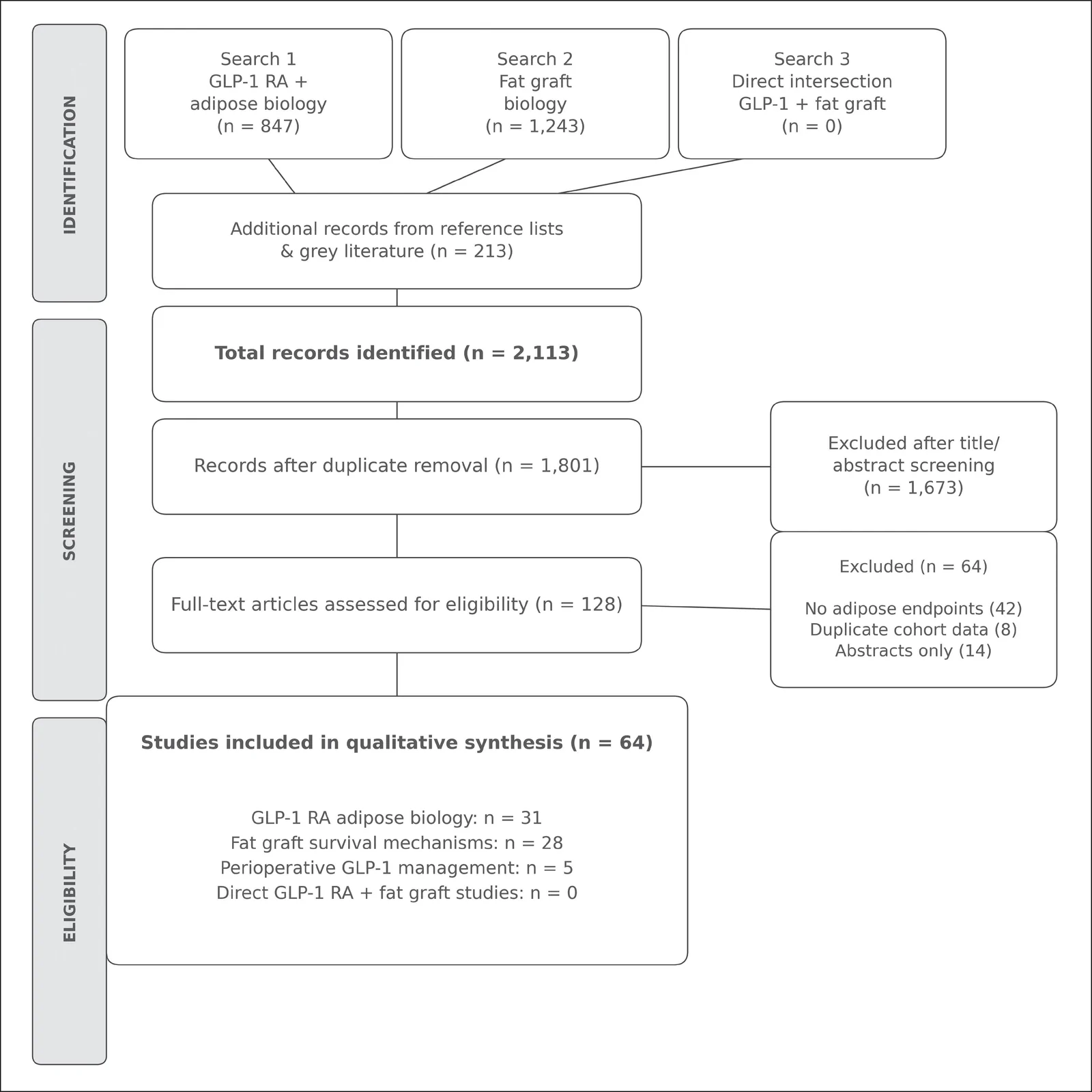
PRISMA-ScR flow diagram. Flow diagram illustrating the systematic search strategy and study selection process for the scoping review. Three independent searches were conducted across PubMed/MEDLINE, Embase, and Google Scholar targeting GLP-1 receptor agonist adipose biology (Search 1; *n* = 847), fat graft survival biology (Search 2; *n* = 1243), and the direct intersection of GLP-1 receptor agonists and fat grafting (Search 3; *n* = 0). An additional 213 records were identified from reference lists and gray literature. After duplicate removal, 1801 records were screened, 128 full-text articles were assessed for eligibility, and 64 studies were included in the qualitative synthesis. Categories of included studies: GLP-1 RA adipose biology (*n* = 31), fat graft survival mechanisms (*n* = 28), perioperative GLP-1 management (*n* = 5), and direct GLP-1 RA + fat graft studies (*n* = 0). PRISMA-ScR indicates Preferred Reporting Items for Systematic Reviews and Meta-Analyses extension for Scoping Reviews.

### Study Selection and Data Synthesis

Eligibility criteria were defined a priori. For inclusion, articles were required to report on at least one of: (1) GLP-1 RA effects on adipocyte biology, browning, or lipolysis; (2) GLP-1 RA effects on ASC differentiation or function; (3) GLP-1 RA effects on adipose tissue remodeling in vivo; or (4) fat graft survival mechanisms including revascularization and adipogenesis. Both preclinical (in vitro, animal) and clinical study designs were eligible, including randomized controlled trials, cohort studies, case series, and mechanistic studies. Systematic reviews and narrative reviews were included for contextual background. No language or date restrictions were applied. Exclusion criteria comprised: conference abstracts without full-text data, studies examining GLP-1 effects exclusively on nonadipose tissues (eg, liver, pancreas) without adipose endpoints, and studies on fat grafting that did not address survival mechanisms. Titles and abstracts were screened by 2 reviewers independently, with disagreements resolved by consensus. Full-text articles were assessed for eligibility against the predefined criteria. Data were charted using a standardized extraction form recording study design, GLP-1 RA type, adipose tissue endpoint(s), species/model, and key findings. Data were synthesized qualitatively, mapping mechanistic effects of GLP-1 RAs onto established models of fat graft biology to identify potential interference points. As this is a scoping review, formal meta-analysis was not performed; however, the level and type of evidence for each interference point is explicitly reported in Table 2.

### Limitations of Search Strategy

Despite a comprehensive search strategy, several limitations should be noted. Studies indexed under alternative terminology (eg, “biostimulation,” “regenerative lipotransfer,” or proprietary drug names not included in our search) may have been missed. The direct intersection search (Search 3) returned zero results, confirming that no study has directly examined GLP-1 RA effects on fat graft outcomes; consequently, the evidence synthesized herein is necessarily indirect, derived from 2 separate bodies of literature that have never been directly connected. This scoping review was not registered prospectively on PROSPERO; we note that PROSPERO does not currently accept scoping review registrations, though protocols can be registered on the Open Science Framework (OSF). All conclusions regarding the impact of GLP-1 RAs on fat graft survival are therefore indirect inferences rather than observed clinical outcomes, and should be treated as exploratory rather than confirmatory (Figure 1).

## RESULTS

### Glucagon-Like Peptide-1 Receptor Agonist (GLP-1 RA) Pharmacology and Adipose Tissue Effects

#### GLP-1 Receptor Expression and Signaling in Adipose Tissue

GLP-1 receptors (GLP-1Rs, cell-surface proteins that bind GLP-1 and initiate intracellular signaling) have been identified in adipose tissue, where they belong to the class B family of G protein-coupled receptors.^6,7^ In subcutaneous and visceral fat depots, GLP-1R activation triggers cAMP/PKA signaling cascades (intracellular messenger pathways that regulate fat metabolism), initiating a range of metabolic effects. The following evidence is drawn predominantly from in vitro studies and limited human biopsy data; the clinical significance of these findings in the context of fat grafting remains to be established.

#### Semaglutide and Liraglutide: GLP-1 Receptor Monoagonism

Semaglutide (half-life ∼7 days, once-weekly dosing) and liraglutide (half-life ∼13 hours, daily dosing) both activate GLP-1 receptors and produce convergent effects on adipose tissue. In a study of obese patients, 6 months of semaglutide therapy reduced adipocyte cross-sectional area, increased UCP1 expression and multilocular (“brown-like”) morphology in subcutaneous fat biopsy specimens, and upregulated genes for lipolysis, fatty acid uptake, and mitochondrial biogenesis (in vivo human data).^6^ Semaglutide also enhanced ASC proliferation and shifted their differentiation toward thermogenic beige adipocytes rather than classical white adipocytes.^6^ In human Simpson-Golabi-Behmel syndrome (SGBS) adipocytes (a validated cell line for studying human fat cell biology), liraglutide induced browning characterized by smaller lipid droplets, upregulation of UCP1, and increased expression of ATGL and HSL.^8^ Taken together, GLP-1 receptor activation shrinks mature adipocytes and reprograms both adipocytes and progenitor cells toward a fat-burning, thermogenic phenotype, raising fundamental questions about the suitability of GLP-1 RA-exposed adipose tissue as graft material.^7,8^

#### Tirzepatide: Dual GLP-1/Glucose-Dependent Insulinotropic Polypeptide (GIP) Receptor Agonism

Tirzepatide simultaneously activates both GLP-1 and glucose-dependent insulinotropic polypeptide (GIP, a gut hormone that also influences fat metabolism) receptors. The TABFAT trial, a randomized placebo-controlled study currently underway, aims to quantify beige adipocyte induction in subcutaneous white adipose tissue biopsies from patients receiving tirzepatide.^12^ In preclinical models, tirzepatide increases UCP1 levels alongside respiratory complex subunits and enhances mitochondrial mass.^13^

#### Retatrutide: Triple GLP-1/GIP/Glucagon Receptor Agonism

Retatrutide (LY3437943) is a first-in-class triple receptor agonist whose addition of glucagon receptor agonism introduces a qualitatively different dimension of adipose tissue modulation. Glucagon acts directly on adipose tissue to decrease lipogenesis and induce lipolysis, and in brown adipose tissue (BAT, metabolically active fat specialized for heat generation) stimulates thermogenesis through UCP1-dependent and FGF21-dependent pathways.^14^ Phase 2 data demonstrated up to 24.2% mean weight loss after 48 weeks, and phase 3 TRIUMPH-4 data showed 28.7% average weight loss (∼71 lbs)—the largest weight loss recorded for any obesity medication.^9,10^

Of particular relevance to the aesthetic surgery community is the growing underground use of retatrutide in bodybuilding and fitness communities. Despite lacking regulatory approval, retatrutide is being sourced from gray-market peptide suppliers and used for body recomposition during cutting phases.^15^ Counterfeit preparations have been documented in the United Kingdom for as little as £2 per dose.^16^ Aesthetic surgeons should anticipate encountering patients who may not disclose use of these unregulated compounds. These effects are summarized in Table 1.

**Table 1.** Summary of GLP-1 RA Effects on Adipose Tissue Biology Relevant to Fat Grafting

Drug class	Adipocyte effects	ASC effects	References
Semaglutide (GLP-1)	UCP1 upregulation; reduced adipocyte cross-sectional area; multilocular morphology; enhanced lipolysis and FA oxidation	Enhanced ASC proliferation; preferential differentiation toward beige adipocytes over white	Szekeres et al^6^
Liraglutide (GLP-1)	Smaller lipid droplets; ATGL/HSL upregulation; UCP1 induction; improved mitochondrial function	Not directly studied in ASCs to date	Bu et al^8^
Tirzepatide (GLP-1/GIP)	UCP1 increase; respiratory complex upregulation; enhanced mitochondrial mass (TOM20)	TABFAT trial underway; results pending	Herman et al^12^; Ma et al^13^
Retatrutide (GLP-1/GIP/GCGR)	All GLP-1 effects plus glucagon-mediated lipolysis; BAT thermogenesis via UCP1 and FGF21; 28.7% weight loss	No ASC-specific data; inferred from GLP-1 and glucagon biology	Jastreboff et al^9^; Habegger et al^14^

ASC, adipose-derived stem cell; ATGL, adipose triglyceride lipase; BAT, brown adipose tissue; FA, fatty acid; FGF21, fibroblast growth factor 21; GCGR, glucagon receptor; GIP, glucose-dependent insulinotropic polypeptide; GLP-1, glucagon-like peptide-1; HSL, hormone-sensitive lipase; RA, receptor agonist; UCP1, uncoupling protein 1.

### Mechanisms of Fat Graft Survival

Nonvascularized fat grafts undergo a predictable biological cascade following transfer, with 3 distinct concentric zones determining graft fate.^4,11^ The survival zone (approximately 200-300 μm from the graft periphery) maintains sufficient oxygen diffusion by plasmatic imbibition (absorption of surrounding fluid that nourishes cells before blood vessels form) to support both mature adipocyte and ASC viability.^4^ The regenerating zone (approximately 600-1200 μm) loses its original adipocytes but retains ASCs, which proliferate, undergo adipogenic differentiation, and secrete angiogenic factors (VEGF, HGF) that promote capillary ingrowth.^17-19^ The necrotic zone at the graft center represents tissue where both adipocytes and ASCs succumb to prolonged ischemia.^20^

Final graft volume depends on (1) how many original adipocytes survive initial ischemia; (2) how effectively new vessels form and (3) how well ASCs regenerate stable white fat.^4,21^ Enriching grafts with additional ASCs or stromal vascular fraction (SVF, the mixed cell population isolated from fat tissue that includes stem cells, immune cells, and vascular precursors) has been shown to improve retention.^19^ Published retention rates range from 46% to 58% in breast augmentation and 30% to 83% in facial applications.^22,23^ Critically, prospective MRI data indicate that weight change is a powerful determinant of retention: patients losing even 1 BMI point postoperatively demonstrated retention of only 22%, compared with 57% and 85% in those gaining 1 and 2 BMI points, respectively (level II evidence).^24^

### Potential Interference Points: Mapping GLP-1 RA Biology Onto Fat Graft Survival

The following analysis overlays the documented effects of GLP-1 RAs on adipose tissue biology with the established mechanisms of fat graft take. It is important to emphasize that each interference point represents a mechanistic hypothesis derived from indirect evidence; none has been validated in a fat grafting model. The strength and clinical significance of each mechanism remain unknown.

#### Adipocyte Browning and Thermogenic Death

In vitro and human biopsy data indicate that GLP-1 RAs can induce UCP1 expression and mitochondrial uncoupling in white fat, reprograming energy-storing adipocytes toward an energy-dissipating phenotype.^6-8^ Hypothetically, a graft droplet containing such “browned” adipocytes could actively oxidize its triglycerides to produce heat. Under the ischemic conditions inherent to nonvascularized fat transfer, this heightened metabolic demand might outstrip available oxygen supply, potentially hastening adipocyte apoptosis, a phenomenon we term “metabolic burn-out.” Rather than passively surviving by low-rate diffusion, graft adipocytes exposed to GLP-1 RAs may consume their own energy stores.^6^ It must be emphasized that this mechanism has not been tested in any fat grafting model; it remains a reasonable but unvalidated hypothesis.

The theoretical risk may be amplified with retatrutide, whose glucagon receptor agonism independently stimulates thermogenesis in both brown and white adipose tissue through UCP1-dependent and FGF21-dependent pathways (preclinical data).^14^ The convergence of GLP-1-mediated browning, GIP-mediated metabolic remodeling, and glucagon-mediated thermogenesis creates a hypothetical “triple threat” to white adipocyte energy storage in grafted tissue. However, whether these effects are additive, synergistic, or partially redundant in vivo remains entirely unknown.

#### Enhanced Lipolysis and Volume Depletion

GLP-1 RAs upregulate the lipolytic enzymes ATGL and HSL and activate AMPK in adipose tissue, promoting triglyceride breakdown and adipocyte shrinkage.^8^ In obese patients, 6 months of semaglutide therapy measurably reduced adipocyte cross-sectional area.^6^ A fat graft in a patient on active GLP-1 RA therapy may therefore lose volume not solely through ischemic cell death but also through pharmacologically driven lipid depletion within surviving cells. This mechanism is distinct from and potentially additive to the browning/thermogenesis pathway.

The clinical relevance is underscored by prospective MRI data demonstrating that postoperative weight loss dramatically reduces fat graft retention.^24^ Patients on GLP-1 RAs are, by definition, in a state of ongoing negative energy balance and adipose tissue catabolism. Whether the systemic metabolic milieu of active GLP-1 RA therapy creates an inhospitable environment for fat graft survival, even after the patient's weight has stabilized on therapy, remains an unanswered question.

#### Altered Adipose-Derived Stem Cell Fate

Adipose-derived stem cells are central to fat graft survival, functioning through both direct adipocyte replacement in the regenerating zone and paracrine signaling that promotes angiogenesis.^18,19^ Recent in vitro evidence indicates that semaglutide-treated human ASCs demonstrate enhanced proliferation but preferential differentiation into thermogenic beige adipocytes rather than lipid-storing white adipocytes.^6^ If this effect translates to the in vivo setting, ASCs responsible for replacing ischemic adipocytes in the regenerating zone could rebuild graft volume with metabolically active fat that is inherently lower in lipid content and higher in energy expenditure, rather than the stable, inert white fat that is the desired endpoint of successful grafting. This remains speculative.

It should be noted that this evidence derives from in vitro studies and short-term in vivo observations. Whether ASC differentiation bias persists after drug washout, and at what time point it reverses, has not been established. Additionally, factors known to improve graft take (such as hypoxia preconditioning, platelet-rich plasma, and ASC enrichment) work by reducing catabolism or boosting stem cell-driven angiogenesis and adipogenesis,^19^ essentially the opposite of GLP-1 RA action on ASCs.

#### Modulation of Angiogenesis and Inflammation

Revascularization is the rate-limiting step in fat graft survival, and the early inflammatory response serves a critical function in recruiting vascular ingrowth and stimulating ASC activation.^11^ Glucagon-like peptide-1 receptor agonists exert complex and potentially contradictory effects on these processes. In vitro and observational data suggest they reduce proinflammatory adipokines and endoplasmic reticulum stress in fat tissue, which is generally metabolically favorable but could theoretically dampen the early inflammatory signals that normally recruit ASCs and promote angiogenesis in grafted fat.^6^

Conversely, some evidence suggests GLP-1 receptor activation may promote endothelial function and nitric oxide production, potentially aiding perfusion.^25^ The net impact on fat graft revascularization is therefore uncertain, and it is not possible to predict from the available evidence whether the proangiogenic or procatabolic effects would predominate in the specific context of a transplanted fat graft. This question can only be resolved by direct experimental investigation (Table 2).

**Table 2.** Potential Interference Points: GLP-1 RA Effects Mapped Onto Fat Graft Survival Mechanisms

Interference point	Mechanism	Graft phase affected	Evidence level
Adipocyte browning	UCP1 upregulation; mitochondrial uncoupling; thermogenic energy dissipation	Survival zone: increased metabolic demand under ischemia	In vitro and in vivo (human biopsy) (6, 8)
Enhanced lipolysis	ATGL/HSL upregulation; AMPK activation; reduced adipocyte size	Survival and regenerating zones: progressive volume depletion	In vitro (human adipocytes) (8); in vivo (6)
ASC fate redirection	Preferential beige over white adipogenesis; reduced PPAR-γ/LPL/AP2	Regenerating zone: impaired white adipocyte replacement	In vitro (human ASCs) (6)
Attenuated inflammation	Reduced proinflammatory adipokines; ER stress reduction	Revascularization: potentially blunted angiogenic signaling	Observational; mechanistic inference (6)
Glucagon-mediated thermogenesis (retatrutide)	Additional lipolysis and BAT activation via UCP1/FGF21 pathways	All zones: amplified catabolic stress	Preclinical (animal models) (14)

AMPK, AMP-activated protein kinase; ASC, adipose-derived stem cell; ATGL, adipose triglyceride lipase; BAT, brown adipose tissue; ER, endoplasmic reticulum; FGF21, fibroblast growth factor 21; GLP-1, glucagon-like peptide-1; HSL, hormone-sensitive lipase; LPL, lipoprotein lipase; PPAR-γ, peroxisome proliferator-activated receptor gamma; RA, receptor agonist; UCP1, uncoupling protein 1.

### The Retatrutide Question: Triple Agonism and the Bodybuilding Demographic

Retatrutide warrants specific attention for several reasons. First, its glucagon receptor agonism introduces a qualitatively distinct mechanism of adipose tissue catabolism that operates independently of GLP-1 signaling, adding direct lipolysis and thermogenesis through pathways not engaged by semaglutide or tirzepatide (preclinical data).^14^ Whether the combined effects of simultaneously activating all 3 receptor pathways are additive, synergistic, or partially redundant in human adipose tissue has not been determined.

Second, retatrutide achieves weight loss of a magnitude previously seen only with bariatric surgery: a 28.7% mean body weight reduction (level I evidence from RCTs).^10^ Whether adipose tissue that has undergone such profound pharmacological remodeling retains the capacity to serve as viable graft material is an open question. Reductions in leptin levels at higher doses reflect progressive depletion of adipose tissue stores, while increases in adiponectin are consistent with fundamental changes in adipose tissue composition.^26^

Third, and most relevant to practicing aesthetic surgeons, retatrutide is being sourced and used outside of clinical trial settings by bodybuilding and fitness communities for aggressive fat loss during cutting phases, with gray-market peptide suppliers offering research-grade compounds of uncertain purity and potency.^15,16^ These individuals may subsequently present for aesthetic procedures (including fat transfer to address localized volume deficits) without disclosing their use of unregulated peptides. The aesthetic surgeon's consultation should therefore include directed, nonjudgmental questioning about peptide and research compound use, particularly in younger, fitness-oriented patients.

## DISCUSSION

### Clinical Considerations

No formal guidelines exist for perioperative management of GLP-1 RAs in fat transfer patients, and the following considerations are based entirely on indirect reasoning and analogy to existing perioperative protocols. They should be understood as speculative rather than evidence-based recommendations and should be revised as clinical data become available.

Regarding the timing of GLP-1 RA cessation, current anesthesiology guidelines recommend withholding daily GLP-1 RAs 1 day before elective procedures and weekly agents approximately 1 week prior to mitigate aspiration risk from delayed gastric emptying.^27^ However, the adipose tissue effects may persist considerably longer than pharmacokinetic half-lives suggest. Semaglutide's 7-day half-life means clinically significant drug levels persist for 5 to 6 weeks after the last dose. The tissue-level effects (browning, altered ASC differentiation, enhanced lipolytic enzyme expression) may require additional time to reverse. A longer washout period (potentially 8-12 weeks) before planned fat transfer could be considered, though this recommendation is speculative, requires clinical validation, and must be weighed against the risks of weight regain and metabolic deterioration.

Regarding weight stability, given that prospective data suggest even modest postoperative weight loss (1 BMI point) reduces fat graft retention to 22%,^24^ performing fat transfer in a patient who is actively losing weight on pharmacotherapy may yield suboptimal results. A minimum of 6 months of demonstrated weight stability on a stable drug dose could be considered before fat grafting, though no data exist to validate this specific timeframe. One approach that has been proposed would be to perform the fat transfer, then reintroduce the GLP-1 RA after 2 to 3 months once graft integration is underway—though this strategy remains entirely untested.

Regarding donor site selection, adipose tissue that has undergone significant browning and metabolic remodeling may contain a higher proportion of thermogenically active adipocytes less suitable for grafting. Subcutaneous depots that are relatively resistant to browning, such as the flanks and lateral thighs, may be preferable, though depot-specific responses to GLP-1 RAs in the context of fat grafting have not been studied.

Regarding ASC supplementation, cell-assisted lipotransfer or stromal vascular fraction (SVF) enrichment may theoretically be valuable in GLP-1 RA-exposed patients, given that SVF enrichment has improved graft retention in non-GLP-1 RA contexts.^19^ Whether this benefit is preserved when ASCs have been chronically exposed to GLP-1 RA signaling, and may therefore be biased toward beige rather than white adipogenesis, requires investigation.^6^

Finally, informed consent should explicitly document GLP-1 RA use and acknowledge the theoretical risks. Surgeons should consider measuring graft volume objectively (eg, with 3D photography, ultrasound, or MRI) during follow-up to contribute prospective data to this emerging question.

### Limitations

This review has several important limitations that must be acknowledged. First, although this scoping review was conducted following PRISMA-ScR guidelines, the protocol was not registered prospectively on PROSPERO; we note that PROSPERO does not currently accept scoping review registrations, though the protocol could have been registered on the OSF. The exploratory nature of mapping 2 disconnected literatures limits the reproducibility of study selection decisions. Relevant studies indexed under alternative search terms may have been missed. Second, and most critically, no study has directly examined the effect of GLP-1 RA administration on fat graft survival in any model—animal or human. All conclusions are mechanistic inferences derived from 2 separate bodies of literature (incretin biology and fat graft science) that have never been directly connected. The strength and direction of each proposed interference point in an actual fat graft remain entirely unknown. Third, the evidence on GLP-1 RA effects in adipose tissue is itself largely preclinical, with limited human biopsy data and no long-term adipose tissue studies in the context of surgical procedures. Fourth, we have necessarily focused on the potentially deleterious effects of GLP-1 RAs on fat graft biology; it is reasonable that some effects (eg, improved vascular function, reduced inflammation) could partially offset the catabolic mechanisms. Fifth, the evidence base for fat graft retention rates is itself characterized by marked heterogeneity in technique, assessment methodology, and follow-up duration, making baseline comparisons challenging. Finally, the clinical recommendations offered are expert opinion at best (level V evidence) and should not be interpreted as guidelines.

### Future Directions

This topic demands a structured research program. Preclinical studies should establish proof-of-concept using validated murine fat grafting models with concurrent GLP-1 RA administration, measuring volumetric retention by micro-CT, histological browning markers (UCP1, CIDEA) in grafted tissue, ASC differentiation profiles, and vascular density by CD31 immunostaining. In vitro experiments could examine human fat graft explants cultured with GLP-1 RAs to determine whether adipocytes consume their lipid stores and whether ASC differentiation is altered under these conditions.

Retrospective clinical studies should compare fat graft outcomes in patients with and without GLP-1 RA history, utilizing objective 3D volumetric imaging at standardized time points (1, 3, 6, and 12 months). Subgroup analyses should stratify by drug class, duration of therapy, washout period, and concurrent vs discontinued use. Dose–response relationships and drug-specific comparisons (mono- vs dual- vs triple-agonist) should be investigated to determine whether the magnitude of adipose tissue effects correlates with the degree of receptor engagement. Optimal washout periods for each drug class warrant specific investigation, given the differing pharmacokinetic profiles and tissue-level persistence of these agents. A prospective registry or survey of aesthetic surgeons for anecdotal evidence of altered graft take in GLP-1 RA users would also be valuable.

Ultimately, a prospective randomized trial (pause vs continue GLP-1 RA) with objective volumetric endpoints could establish clear guidelines. Given the rapid evolution of this drug class (with survodutide, pemvidutide, orforglipron, and other agents in late-stage development), the aesthetic surgery community must develop a responsive research framework that can evaluate new agents as they reach the market.

## CONCLUSIONS

The coincidence of widespread GLP-1 RA adoption and the increasing demand for autologous fat grafting represents one of the most pressing unanswered questions in contemporary aesthetic surgery. The mechanistic evidence reviewed herein, drawn from preclinical adipose tissue studies and established fat graft biology, provides a plausible biological rationale for concern: GLP-1 RAs promote adipocyte browning and thermogenic activation, enhance lipolysis through upregulation of ATGL and HSL, redirect ASC differentiation toward beige lineages, and modulate the inflammatory responses that may be important for graft revascularization. The emergence of retatrutide as a triple agonist amplifies these concerns, while its underground adoption introduces an undisclosed exposure variable.

However, it must be clearly stated that the clinical significance of these mechanisms for fat graft outcomes has not been demonstrated. It is possible that the proangiogenic and anti-inflammatory properties of GLP-1 RAs partially or wholly offset their catabolic effects. It is equally possible that fat graft failure in GLP-1 RA-treated patients is already occurring and being attributed to other variables. Until rigorous preclinical and clinical data are available, we suggest that aesthetic surgeons performing fat transfer in patients with current or recent GLP-1 RA exposure should document drug use, counsel patients regarding the theoretical uncertainty, consider the perioperative timing of drug cessation, and—most importantly, collect and publish outcome data to begin building the evidence base that this rapidly expanding patient population urgently needs.

## Supplemental Material

This article contains supplemental material located online at https://doi.org/10.1093/asj/sjag108.

## Supplementary Material

sjag108_Supplementary_Data
