# Supplementary material for: Do Glucagon-Like Peptide-1 Receptor Agonists Sabotage Fat Grafts?: A Scoping Review of Glucagon-Like Peptide-1 Receptor Agonist Effects on Adipocyte Biology and Implications for Autologous Fat Transfer
Source: Aesthet Surg J. 2026 Jun 1;46(11):1214–20. doi: 10.1093/asj/sjag108 (PMC13668360; doi:10.1093/asj/sjag108)
Supplement: Supplementary file 1 [file sjag108_supplementary_data.docx]

**Supplemental Table 1**. Glossary of Key Terms

| Term / Abbreviation | Definition |
| --- | --- |
| Adipocyte | A fat cell; the primary cell type in adipose (fat) tissue that stores energy as triglycerides (fat molecules) |
| Adipose-derived stem cell (ASC) | A type of adult stem cell found within fat tissue that can develop into new fat cells, support blood vessel growth, and secrete growth factors important for tissue repair |
| AMPK (AMP-activated protein kinase) | An enzyme that acts as a cellular energy sensor; when activated, it promotes fat breakdown and energy production |
| Angiogenesis | The formation of new blood vessels from pre-existing vessels; critical for fat graft survival as it restores blood supply to transplanted tissue |
| ATGL (adipose triglyceride lipase) | The enzyme that initiates the first step of fat breakdown (lipolysis) inside fat cells by removing fatty acids from stored triglycerides |
| Beige/brown adipocyte | Fat cells specialized for burning energy as heat rather than storing it; beige cells arise within white fat depots, while brown fat exists in dedicated depots |
| Browning | The process by which white (energy-storing) fat cells are converted into beige (energy-burning) fat cells, typically involving UCP1 upregulation |
| cAMP/PKA signaling | An intracellular communication pathway (cyclic AMP / protein kinase A) that relays signals from cell-surface receptors to trigger metabolic changes inside cells |
| Cell-assisted lipotransfer (CAL) | A fat grafting technique in which the graft is enriched with additional stem cells (ASCs) to improve survival |
| FGF21 (fibroblast growth factor 21) | A hormone-like protein that promotes fat burning, improves metabolism, and is involved in glucagon-mediated thermogenesis |
| GIP (glucose-dependent insulinotropic polypeptide) | A gut hormone, like GLP-1, that stimulates insulin release and has distinct effects on fat tissue metabolism |
| GLP-1 (glucagon-like peptide-1) | A gut hormone that suppresses appetite, stimulates insulin release, and has recently been found to affect fat cell biology directly |
| GLP-1 receptor agonist (GLP-1 RA) | A medication that mimics the action of GLP-1 by binding to its receptor, used for diabetes and weight management (e.g., semaglutide, liraglutide) |
| HSL (hormone-sensitive lipase) | An enzyme that performs the second step of fat breakdown inside cells, continuing the process initiated by ATGL |
| Incretin | A class of gut hormones (including GLP-1 and GIP) that are released after eating and help regulate blood sugar and metabolism |
| Lipolysis | The breakdown of stored fat (triglycerides) into fatty acids and glycerol, releasing stored energy |
| Multilocular morphology | A microscopic appearance of fat cells containing many small lipid droplets (typical of beige/brown fat) rather than one large droplet (typical of white fat) |
| Paracrine signaling | Communication between nearby cells through secreted molecules; ASCs support graft survival partly by secreting growth factors that act on neighboring cells |
| Plasmatic imbibition | The passive absorption of surrounding plasma (fluid) by transplanted tissue in the first 24–48 hours, providing nutrients and oxygen before blood vessels grow in |
| PPAR-γ (peroxisome proliferator-activated receptor gamma) | A master regulator protein that controls the development and function of white fat cells; its suppression by GLP-1 RAs may impair normal fat cell formation |
| Stromal vascular fraction (SVF) | The mixed population of cells obtained when fat tissue is processed, including stem cells, immune cells, and blood vessel precursor cells—but not mature fat cells |
| Thermogenesis | The production of heat by the body; in adipose tissue, thermogenesis occurs when fat cells uncouple energy storage from ATP production, dissipating energy as heat |
| UCP1 (uncoupling protein 1) | A mitochondrial protein found in brown and beige fat cells that “uncouples” energy production, causing stored fat to be burned as heat rather than stored |
| VEGF / HGF | Vascular endothelial growth factor / hepatocyte growth factor—proteins secreted by stem cells that stimulate new blood vessel formation (angiogenesis) |
